# A comparative analysis of parametric survival models and machine learning methods in breast cancer prognosis

**DOI:** 10.1038/s41598-025-15696-0

**Published:** 2025-08-25

**Authors:** Sonia Kaindal, B. Venkataramana

**Affiliations:** Department of Mathematics, School of Advanced Science, Vellore Institute of Technology, Vellore, 632014 Tamil Nadu India

**Keywords:** Survival probability, Probability density function, Accuracy, Hazard ratio, Cancer, Diseases, Medical research, Mathematics and computing

## Abstract

Accurate prediction of breast cancer survival is critical for optimizing treatment strategies and improving clinical outcomes. This study evaluated a combination of parametric statistical models and machine learning algorithms to identify the most influential prognostic factors affecting the survival of patients. Two commonly used parametric models, log-gaussian regression and logistic regression, were applied to assess the relationship between survival and a set of clinical variables, including age at diagnosis, tumor grade, primary tumor site, marital status, American Joint Committee on Cancer (AJCC) stage, race, and receipt of radiation therapy or chemotherapy. Machine learning methods, such as neural networks, support vector machines (SVMs), random forests, gradient boosting machines (GBMs), and logistic regression classifiers, were employed to compare the predictive performance. Among these, the neural network model exhibited the highest predictive accuracy. The random forest model achieved the best balance between model fit and complexity, as indicated by its lowest akaike information criterion and bayesian information criterion values. Across all models, five variables consistently emerged as significant predictors of survival: age, tumor grade, ajcc stage, marital status, and radiation therapy use. These findings highlight the importance of combining traditional survival analysis techniques with machine learning approaches to enhance predictive accuracy and support evidence-based personalized treatment planning in breast cancer care.

## Introduction

Breast cancer is a complex and heterogeneous disease that remains a major global health concern, contributing significantly to cancer-related mortality in women^1^. Invasive lobular carcinoma (ILC), which accounts for 10–15% of all breast cancers, is the second most common histological subtype^2^. ILC differs from invasive ductal carcinoma (IDC) in terms of its molecular and biological characteristics^3^. Despite advancements in screening and treatment that have improved overall survival, predicting individual patient outcomes remains challenging and complicates personalized care. Accurate survival prediction is essential for effective risk stratification, informed therapeutic decision-making, and efficient allocation of health care resources. There is a growing need for patient-centered approaches that promote rational and equitable cancer care in oncology^4^.

Survival analysis offers a statistical framework for modeling time-to-event data, which is particularly relevant in oncology, where events such as recurrence, metastasis, and death occur at variable times across patients. The two key components of this framework are the survival function, which estimates the probability of survival beyond a given time, and the hazard rate, which measures the instantaneous risk of an event occurring at a specific time^5^. However, the application of parametric survival models in routine cancer research remains challenging because of their underlying assumptions and complexity^6^. A recent study proposed a deep-learning-based breast cancer diagnosis model enhanced by a hybrid rule-based feature selection technique. Using the wisconsin breast cancer dataset (WBCD), the model identified five key diagnostic features and achieved 99.5% accuracy. By eliminating irrelevant data, the model improved prediction performance and demonstrated superior diagnostic accuracy compared with existing models, indicating a strong potential for early and precise breast cancer detection^7^.

Although traditional parametric models are widely used in breast cancer survival analysis, their limited flexibility in handling nonlinear and high-dimensional data raises concerns regarding predictive accuracy. Conversely, machine learning (ML) methods offer improved predictive performance but often lack clinical interpretability. Therefore, a systematic comparison of these approaches is needed to identify models that best balance accuracy and interpretability for real-world clinical use in breast cancer prognosis.

**Fig. 1:**
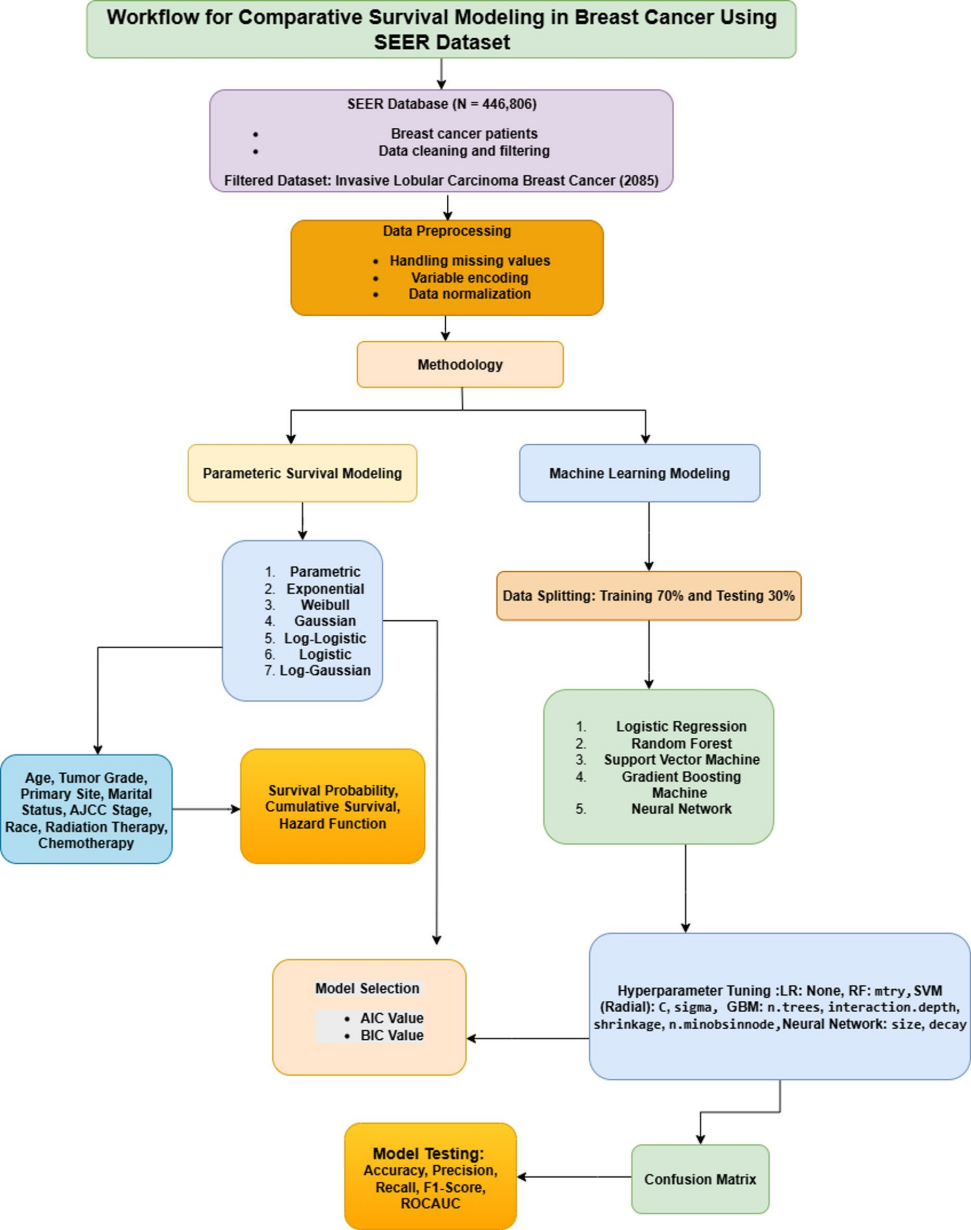
Flow Diagram of Data Analysis and Methodology.

Figure 1 illustrates the methodology for analyzing 2,085 cases of invasive lobular carcinoma from the SEER database (2011 to 2015) using two parallel approaches: parametric survival modeling and machine learning. Survival analysis was performed using exponential, weibull, and log-logistic models to estimate survival probabilities and hazard functions based on clinical variables. Machine learning models, including logistic regression, random forest, support vector machine, gradient boosting, and neural networks, were trained on 70% of the dataset and validated using the remaining 30%. The model performance was evaluated using confusion matrices and metrics such as accuracy, precision, recall, F1 score, and area under the receiver operating characteristic curve.

This study presents a comprehensive analysis of invasive lobular carcinoma (ILC) prognosis using advanced machine-learning techniques. Section “Related work” reviews the existing literature on ILC prediction, highlighting previous methodologies and key findings. Sections “Data design and preprocessing” and “Methodology” describe the data collection process and methodological framework, including details on the dataset, its source, features, and correlation analysis. Section “Experimental results” discusses the experimental results and model comparisons. Section “Conclusion” concludes the study and suggests directions for future research.

## Related work

Theophilus Gyedu Baidoo and Hansapani^8^ evaluated both survival-specific and machine learning models using performance metrics such as the concordance index (c-index), integrated brier score (IBS), and area under the curve (AUC). The cox proportional hazards (CPH) model, random survival forest (RSF), and deepsurv demonstrated strong performance, with RSF achieving a c-index of 0.72. Both cox and RSF recorded the lowest IBS value of 0.08. However, while machine learning models such as random forest (AUC 0.74) and xgboost (AUC 0.69) showed moderate discrimination, they lacked mechanisms for handling censored data, a key limitation in survival analysis. In a related study, the authors applied five machine learning classifiers using 13 selected features, with LightGBM optimized via a tree-structured parzen estimator, achieving 99.86% accuracy, 100% precision, and 99.60% recall, demonstrating high potential in distinguishing between malignant and benign tumors with minimal human intervention^9^.

Jialong Xiao, Miao Mo, et al.^10^ compared machine learning algorithms with the cox model for predicting overall survival in a large breast cancer cohort of 22,176 patients. Their findings revealed that the RSF slightly outperformed the Cox model in terms of discrimination, with a c-index of 0.827 compared to 0.814. This emphasizes the utility of the RSF in prognostic modeling. Another study explored a modified Weibull distribution capable of modeling various hazard rate shapes, including increasing, decreasing, constant, or bathtub-shaped patterns, with results closely aligned with kaplan-meier survival curves^11^. Another study by Tizi and Abdelaziz Berrado^12^ compared machine learning techniques with conventional statistical methods for cancer survival prediction. The study evaluated models, including random survival forests and cox regression with ridge regularization, using the c-index for performance comparison. The results indicated that both approaches performed similarly, although cox regression struggled with high-dimensional data. A separate study applied machine learning models to predict invasive disease-free events in 145 patients, showing that random survival forest with gradient boosting outperformed the cox model (c-index, 0.68 vs. 0.57). These findings suggest that clinical data alone can enhance prediction accuracy and reduce the need for costly genetic testing^13^.

Surbhu Gupta and Manoj K. Gupta^14^ assessed deep learning models, including the restricted boltzmann machine (RBM), for predicting post-operative survival in breast cancer. Using cross-validation, the RBM achieved the highest accuracy (0.97), reinforcing the need for continued evaluation of deep learning architectures for optimal predictive performance.

A study by Sahar A. and El Rahman^15^ investigated early breast cancer detection using machine learning algorithms and feature selection across four datasets. Classifier performance varied across datasets: Random forest with a genetic algorithm achieved 96.82% on WBC, C-SVM with RBF kernel reached 99.04% on WDBC, random forest with recursive feature elimination scored 74.13% on WPBC, and decision tree achieved 83.74%. Another comparative study^16^ reported SVM and LDA achieving 93% accuracy, Random forest 98%, and logistic regression 86%, demonstrating consistent effectiveness across models.

Gunjan et al.^17^, highlighted the importance of early breast cancer detection and reviewed advancements in AI-based computer-aided diagnosis (CAD) systems. They compared machine learning and deep learning approaches with conventional methods, discussing their benefits, limitations, and future directions for medical image analysis. Nermin Abdelhakim Othman et al.^18^ proposed a hybrid deep learning model for predicting breast cancer survival using multi-omics data from the METABRIC dataset. The framework combines a convolutional neural Network CNN-based feature extraction with long short-term memory (LSTM) and gated recurrent unit (GRU) classifiers, achieving an accuracy of 98.0% through decision-level fusion. This model significantly improved survival prediction over single-modality approaches, offering a more robust and accurate tool for personalized breast cancer prognosis.

Another study using the wisconsin breast cancer dataset^19^ evaluated several classifiers, including SVM, k-nearest neighbors, random forest, and logistic regression. SVM emerged as the most accurate, achieving 95% accuracy, reaffirming the role of CAD systems in early detection. A separate comparison of linear and nonlinear models^20^ found that while SVM had higher sensitivity, artificial neural networks offered better overall diagnostic performance, underscoring the value of nonlinear models in complex datasets.

Using Surveillance, Epidemiology, and End Results (SEER) data from 2010 to 2019, a study^21^ developed an xgboost model to predict survival in patients with bone metastatic breast cancer (BMBC). The model achieved AUC scores above 0.79. Prognostic factors such as treatment delays and income levels were significant, with neoadjuvant chemotherapy plus surgery improving outcomes in select subgroups.

Jain et al.^22^ aimed to identify optimal machine learning models for automatic breast cancer diagnosis using the wisconsin dataset. Their results showed that hyperparameter-tuned models and boosting algorithms, such as xgboost, consistently achieved high accuracy for both benign and malignant classifications. A study using the cancer genome atlas - breast invasive carcinoma (TCGA-BRCA) dataset^23^ explored multimodal machine learning systems for survival prediction by integrating six biomedical modalities. Dimensionality reduction techniques and classifiers (SVM, random forest) improved the accuracy and robustness. However, these models lacked prospective validation on primary datasets, indicating the need for real-world testing.

Yinan Huang, Jieni Li, Mai Li, and Rajender R^24^. reviewed 28 studies applying machine learning models to real-world healthcare data for time-to-event outcomes. Random survival forests and neural networks are commonly used in oncology. The review noted the underuse of ML for treatment prediction and emphasized the need for methodological advances to enhance clinical utility.

The study by Chirag Nagpal, Xinyu Li, and Artur Dubrawski^25^ proposed a fully parametric deep learning approach for time-to-event prediction, circumventing the proportional hazards assumption of the Cox model. Their model accurately estimated survival risks in datasets with complex censoring and competing risks, offering a significant advancement in parametric survival modeling. M. Darshan Teja and G. Mokesh Rayalu^26^ utilized University of California, Irvine data to evaluate eight machine learning models for cardiovascular disease prediction. Ensemble methods like random forest and bagged trees achieved the highest accuracy and ROC-AUC. The k-fold validation confirmed model reliability, emphasizing the effectiveness of ensemble techniques in prediction tasks.

Keren Evangeline I., S. P. Angeline Kirubha, and J. Glory Precious^27^ used the METABRIC dataset to identify the predictive variables in breast cancer. They compared the cox proportional hazards (CoxPH) model, RSF, and DeepHit. RSF and DeepHit outperformed CoxPH, both achieving a C-index of 0.86 compared with 0.85 for CoxPH. Key predictors included relapse-free status (RSF), age at diagnosis, estrogen and progesterone receptor status, and tumor stage (cox proportional hazards), aiding clinical decision-making. Recent studies have also focused on enhancing survival prediction through frailty modeling^28^. Another study^29^, revealed that patients in non-manual occupations had better survival (hazard ratio < 0.85), with technicians and associate professionals situated at the manual and non-manual intersection.

A study^30^ employed machine learning to predict survival duration using tumor-related clinical features such as stage, size, and age. Kernel ridge regression, k-nearest neighbors, lasso, and decision tree models demonstrated high predictive accuracy owing to effective data integration techniques. Finally, a study using data from the University of Ilorin Teaching Hospital^31^ applied several machine learning algorithms to predict breast cancer survival. AdaBoost outperformed the other models, achieving 98.3% accuracy and 99.9 AUC, confirming its potential for clinical application.

Although survival analysis has been widely used in breast cancer studies, it has been less studied in the context of invasive lobular carcinoma (ILC). Existing literature commonly employs cox proportional hazards models and random survival forests, with fewer studies examining the performance of other established parametric models, such as weibull, exponential, logistic, log-logistic, gaussian, and log-gaussian distributions. Additionally, the application of formal model selection criteria, such as the akaike information criterion (AIC) and bayesian information criterion (BIC), is less common in studies involving machine-learning approaches. Accordingly, further exploration of diverse modeling techniques and evaluation metrics may contribute to a more comprehensive understanding of survival prediction. This study aims to address this need by comparing multiple parametric and machine learning models for ILC survival prediction, using AIC/BIC and performance metrics to support model evaluation and interpretability in a clinically meaningful context. The objectives of this study were as follows:

1. To investigate the prognostic significance of clinical and pathological factors, such as age, tumor grade, ajcc stage, and treatment, on breast cancer survival outcomes.
2. To conduct a comparative evaluation of parametric survival models and machine learning algorithms in predicting patient survival, utilizing statistical criteria, including AIC, BIC, and ROC-based measures.
3. To identify the most suitable predictive model, we assessed the trade-off between model interpretability and predictive accuracy across various machine learning methods.

## Data design and preprocessing

This study was based on data obtained from the Surveillance, Epidemiology, and End Results (SEER) program, which collects cancer incidence and survival data from population-based registries across the United States. The original dataset included more than 446,000 breast cancer cases. This study focused on patients diagnosed with invasive lobular carcinoma (ILC) between 2011 and 2015, allowing for a more targeted analysis.

To ensure data quality and relevance, patients with missing information on key clinical variables or those diagnosed with other breast cancer subtypes were excluded. After applying these criteria, we identified a final cohort of 2,085 patients for analysis. Each case included information on overall survival time (in months), vital status (alive or deceased), and cause of death, which served as the outcome variables in our analysis. We selected eight clinical features known to influence breast cancer outcomes: age at diagnosis, tumor grade, primary tumor site, marital status, AJCC stage, race, and whether the patients received radiation therapy or chemotherapy. These variables were selected based on their established relevance in previous prognostic studies.

To manage the complexity of the dataset and uncover underlying patterns, we applied principal component analysis (PCA). PCA helped to reduce the dimensionality of the data while preserving the most informative features, making the subsequent modeling process more efficient and interpretable. The dataset was split into training (70%) and testing (30%) subsets for the model. Model development was conducted using the caret package in R, which simplifies the machine learning workflows. We trained and compared five different algorithms: logistic regression, random forest, support vector machine (SVM), gradient-boosting machine (GBM), and neural networks. To improve the model’s reliability and avoid overfitting, we used 10-fold cross-validation during training. Hyperparameter tuning was performed using caret’s tuneLength function, which automatically tests a range of settings to determine the best configuration for each model.

Once the training was complete, the models were evaluated using the testing set. Performance was measured using the following key metrics: accuracy, precision, recall, F1-score, and area under the ROC curve (AUC). Additionally, we used the akaike information criterion (AIC) and bayesian information criterion (BIC), where applicable, to assess model fit and complexity. All data processing, analysis, and visualization were performed using R, with additional tabulations completed in Microsoft Excel.

## Methodology

Parametric survival methods assume that the survival time adheres to a specific probability distribution. These methods calculate the survival functions using probability density functions (PDFs) and cumulative distribution functions (CDFs). We provide six frequently used parametric survival distributions:

### Exponential distribution

The exponential distribution is the simplest survival model, assuming a constant hazard rate over time^32^.

Pdf,

1 $$\:\varvec{f}\left(\varvec{t};\varvec{\lambda\:}\right)=\varvec{\lambda\:}{\varvec{e}}^{-\varvec{\lambda\:}\varvec{t}},\varvec{t}>0,\varvec{\lambda\:}>0$$

Cdf,

2 $$\:\varvec{F}\left(\varvec{t}\right)=1-{\varvec{e}}^{-\varvec{\lambda\:}\varvec{t}},\varvec{t}>0$$

The exponential model indicates that the risk of occurrence remains constant. It is frequently impractical to use medical data when risks fluctuate dynamically.

### Weibull distribution

The Weibull model generalizes the exponential function by allowing a variable hazard rate^33^.

Pdf

3 $$\:\varvec{f}\left(\varvec{t};\varvec{\lambda\:},\varvec{k}\right)=\varvec{k}\varvec{\lambda\:}{\varvec{t}}^{\varvec{k}-1}{\varvec{e}}^{-\varvec{\lambda\:}{\varvec{t}}^{\varvec{k}}},\varvec{t}>0,\varvec{\lambda\:}>0,\varvec{k}>0$$

Cdf

4 $$\:\varvec{F}\left(\varvec{t}\right)=1-{\varvec{e}}^{-\varvec{\lambda\:}{\varvec{t}}^{\varvec{k}}},\varvec{t}>0$$

If k > 1, the hazard function increases over time (useful for the aging process). If k < 1, the hazard decreases over time (useful for early-stage failures). This flexibility makes the weibull distribution widely applicable in survival analyses.

### Logistic distribution

The logistic distribution follows a normal distribution^34^.

Pdf,

5 $$\:\varvec{f}\left(\varvec{t};\varvec{\mu\:},\varvec{s}\right)=\frac{{\varvec{e}}^{-\frac{\varvec{t}-\varvec{\mu\:}}{\varvec{s}}}}{\varvec{s}(1+{\varvec{e}}^{-\frac{\varvec{t}-\varvec{\mu\:}}{\varvec{s}}}{)}^{2}},-\mathbf{\infty\:}<\varvec{t}<\mathbf{\infty\:}$$

Cdf,

6 $$\:\varvec{F}\left(\varvec{t}\right)=\frac{1}{1+{\varvec{e}}^{-\frac{\varvec{t}-\varvec{\mu\:}}{\varvec{s}}}},-\mathbf{\infty\:}<\varvec{t}<\mathbf{\infty\:}$$

The logistic model accounts for symmetric survival time distributions and is used when survival data exhibit heavier tails than the normal distribution.

### Gaussian (Normal) distribution

Normal distribution models the survival time symmetrically around the mean.

Pdf,

7 $$\:\varvec{f}\left(\varvec{t};\varvec{\mu\:},\varvec{\sigma\:}\right)=\frac{1}{\varvec{\sigma\:}\sqrt{2\varvec{\pi\:}}}{\varvec{e}}^{-\frac{(\varvec{t}-\varvec{\mu\:}{)}^{2}}{2{\varvec{\sigma\:}}^{2}}},-\mathbf{\infty\:}<\varvec{t}<\mathbf{\infty\:}$$

Cdf,

8 $$\:\varvec{F}\left(\varvec{t}\right)=\frac{1}{2}\left[1+\mathbf{e}\mathbf{r}\mathbf{f}\left(\frac{\varvec{t}-\varvec{\mu\:}}{\varvec{\sigma\:}\sqrt{2}}\right)\right]$$

The normal distribution is rarely used in survival analysis because it allows negative survival times, which are not meaningful in practice.

### Log-Logistic distribution

The log-logistic model is useful when hazard rates first increase and then decrease over time^35^.

Pdf,

9 $$\:\varvec{f}\left(\varvec{t};\varvec{\alpha\:},\varvec{\beta\:}\right)=\frac{(\varvec{\beta\:}/\varvec{\alpha\:})(\varvec{t}/\varvec{\alpha\:}{)}^{\varvec{\beta\:}-1}}{(1+(\varvec{t}/\varvec{\alpha\:}{)}^{\varvec{\beta\:}}{)}^{2}},\varvec{t}>0,\varvec{\alpha\:}>0,\varvec{\beta\:}>0$$

Cdf,

10 $$\:\varvec{F}\left(\varvec{t}\right)=\frac{1}{1+(\varvec{t}/\varvec{\alpha\:}{)}^{-\varvec{\beta\:}}},\varvec{t}>0$$

This model is useful when the survival time follows a distribution in which the hazard initially increases and then decreases, making it relevant for modeling cancer survival.

### Log-Gaussian (Log-Normal) distribution

The log-normal model is appropriate when survival time follows a skewed distribution.

Pdf,

11 $$\:\varvec{f}\left(\varvec{t};\varvec{\mu\:},\varvec{\sigma\:}\right)=\frac{1}{\varvec{t}\varvec{\sigma\:}\sqrt{2\varvec{\pi\:}}}{\varvec{e}}^{-\frac{(\mathbf{l}\mathbf{n}\varvec{t}-\varvec{\mu\:}{)}^{2}}{2{\varvec{\sigma\:}}^{2}}},\varvec{t}>0$$

Cdf,

12 $$\:\varvec{F}\left(\varvec{t}\right)=\frac{1}{2}\left[1+\mathbf{e}\mathbf{r}\mathbf{f}\left(\frac{\mathbf{l}\mathbf{n}\varvec{t}-\varvec{\mu\:}}{\varvec{\sigma\:}\sqrt{2}}\right)\right]$$

**Table 1:** Survival, hazard, and cumulative hazard functions with interpretations for various survival models.

Model	Survival Function S(t)S(t)	Hazard Function h(t)h(t)	Cumulative Hazard H(t)H(t)
Exponential	$\:{e}^{-\lambda\:t}$	$\:\lambda\:$	$\:\lambda\:t$
Weibull	$\:{e}^{-(\lambda\:t{)}^{k}}$	$\:k{\lambda\:}^{k}{t}^{k-1}$	$\:(\lambda\:t{)}^{k}$
Gaussian (Normal)	$\:\text{(}1-{\Phi\:}\left(\frac{t-\mu\:}{\sigma\:}\right)$	$\:\frac{f\left(t\right)}{S\left(t\right)}=\frac{\frac{1}{\sigma\:\sqrt{2\pi\:}}{e}^{-\frac{(t-\mu\:{)}^{2}}{2{\sigma\:}^{2}}}}{1-{\Phi\:}\left(\frac{t-\mu\:}{\sigma\:}\right)}$	$\:-\text{log}\left(1-{\Phi\:}\left(\frac{t-\mu\:}{\sigma\:}\right)\right)$
Log-Logistic	$\:\frac{1}{1+(\lambda\:t{)}^{k}}$	$\:\frac{k{\lambda\:}^{k}{t}^{k-1}}{1+(\lambda\:t{)}^{k}}$	$\:\text{l}\text{o}\text{g}(1+(\lambda\:t{)}^{k})$
Logistic	$\:\frac{1}{1+{e}^{\frac{t-\mu\:}{\sigma\:}}}$	$\:\frac{{e}^{\frac{t-\mu\:}{\sigma\:}}}{\sigma\:\left(1+{e}^{\frac{t-\mu\:}{\sigma\:}}\right)}$	$\:\text{log}\left(1+{e}^{\frac{t-\mu\:}{\sigma\:}}\right)$
Log-Gaussian (Log-Normal)	$\:1-{\Phi\:}\left(\frac{\text{log}t-\mu\:}{\sigma\:}\right)$	$\:\frac{f\left(t\right)}{S\left(t\right)}=\frac{\frac{1}{t\sigma\:\sqrt{2\pi\:}}{e}^{-\frac{(\text{l}\text{o}\text{g}t-\mu\:{)}^{2}}{2{\sigma\:}^{2}}}}{1-{\Phi\:}\left(\frac{\text{log}t-\mu\:}{\sigma\:}\right)}$	$\:-\text{log}\left(1-{\Phi\:}\left(\frac{\text{log}t-\mu\:}{\sigma\:}\right)\right)$

Table 1 represents the survival function $\:S\left(t\right)\:$, hazard function $\:h\left(t\right)\:$, and the cumulative hazard function $\:H\left(t\right)$ which characterize the properties of various statistical distributions in survival analysis. The exponential model assumes a constant hazard rate, resulting in a straightforward, exponentially declining survival probability curve. The weibull model extends this by introducing a shape parameter k, which allows for an increasing or decreasing hazard rate over time. The gaussian (normal) model characterizes survival using the standard normal cumulative distribution function (cdf), with hazard functions that depend on the corresponding probability density function (pdf). The log-logistic and logistic models generate sigmoid-shaped survival curves determined by their respective scale parameters. Finally, the log-gaussian (log-normal) model applies a logarithmic transformation to survival times, offering flexibility in modeling skewed distributions.

**Confusion Matrix:**

A confusion matrix is an essential classification technique that summarizes predictions with actual results. Table 2 lists these four components.

- False Positives (FP): Incorrectly predicted positive cases.
- True Positives (TP): Correctly predicted positive cases.
- False Negatives (FN): Incorrectly predicted negative cases.
- True Negatives (TN): Correctly predicted negative cases.

**Table 2:** Confusion Matrix.

	Predicted Positive	Predicted Negative
Actual Positive	TP	FN
Actual Negative	FP	TN

### Accuracy

Accuracy is a fundamental metric that denotes the ratio of correctly classified cases to the total occurrences [bustillo2022improving].

13 $$\:\text{Accuracy}=\frac{\varvec{T}\varvec{P}+\varvec{T}\varvec{N}}{\varvec{T}\varvec{P}+\varvec{T}\varvec{N}+\varvec{F}\varvec{P}+\varvec{F}\varvec{N}}$$

### Precision

Precision, or positive predictive value (PPV), quantifies the ratio of accurately identified positive cases to the total projected positive cases.

14 $$\:\text{Precision}=\frac{\varvec{T}\varvec{P}}{\varvec{T}\varvec{P}+\varvec{F}\varvec{P}}$$

Precision is important in scenarios in which false positives must be minimized.

### Recall (sensitivity)

Recall, referred to as sensitivity or true positive rate (TPR), quantifies the ratio of accurately anticipated positive cases to the total number of actual positive cases.

15 $$\:\text{Recall}=\frac{\varvec{T}\varvec{P}}{\varvec{T}\varvec{P}+\varvec{F}\varvec{N}}$$

High recall is crucial in medical applications, where missing a positive case (false negative) is dangerous, such as failing to detect breast cancer in patients.

### F1-score

The F1-score is the harmonic mean of precision and recall, balancing both metrics.

16 $$\:\text{F1-Score}=2\times\:\frac{\text{Precision}\times\:\text{Recall}}{\text{Precision}+\text{Recall}}$$

The F1-score is a useful metric when dealing with imbalanced datasets, as it ensures a balance between precision and recall.

### Area under the curve (AUC)

The AUC is determined from the receiver operating characteristic (ROC) curve, which graphs the true positive rate (recall) versus the false positive rate (FPR)^36^.

17 $$\:\text{False\:Positive\:Rate\:(FPR)}=\frac{\varvec{F}\varvec{P}}{\varvec{F}\varvec{P}+\varvec{T}\varvec{N}}$$

- The AUC signifies the likelihood that the model prioritizes a randomly selected positive occurrence above a randomly selected negative case.
- An AUC of 0.5 indicates a model with no discrimination capability (i.e., random guessing).
- An AUC value close to 1.0 indicates an excellent model.

**Table 3:** Mathematical equations for ML Models.

Model	Equation
Logistic Regression	$\:P\left(Y=1\mid X\right)=\frac{1}{1+{e}^{-\left({\beta\:}_{0}+\sum\:_{i=1}^{n}{\beta\:}_{i}{X}_{i}\right)}}$
Random Forest	$\:y=\frac{1}{T}\sum\:_{t=1}^{T}{f}_{t}\left(X\right)$
	Gini: $\:G=1-\sum\:_{i=1}^{c}{p}_{i}^{2}$
	Entropy: $\:H=-\sum\:_{i=1}^{c}{p}_{i}{\text{log}}_{2}\left({p}_{i}\right)$
Support Vector Machine (SVM)	$\:\underset{w,b}{min}\frac{1}{2}\left|\right|w\left|{|}^{2}\text{s.t.}{y}_{i}\right(w\cdot\:{X}_{i}+b)\ge\:1,\forall\:$
	Kernel Trick: $\:K\left({X}_{i},{X}_{j}\right)={e}^{-\gamma\:\left|\right|{X}_{i}-{X}_{j}|{|}^{2}}$
Gradient Boosting Machine (GBM)	$\:{F}_{m}\left(X\right)={F}_{m-1}\left(X\right)+{\gamma\:}_{m}{h}_{m}\left(X\right)$
	$$\:{\gamma\:}_{m}=\text{arg}\underset{\gamma\:}{min}\sum\:_{i=1}^{n}L\left({y}_{i},{F}_{m-1}\left({X}_{i}\right)+\gamma\:{h}_{m}\left({X}_{i}\right)\right)$$
Neural Network	$\:Z={W}_{1}X+{b}_{1}$
	$$\:A=\sigma\:\left(Z\right)=\frac{1}{1+{e}^{-Z}}$$
	$$\\hat :{y}={W}_{2}A+{b}_{2}$$
	Weight Update: $\:W\leftarrow\:W-\eta\:\frac{\partial\:L}{\partial\:W}$

Table 3 presents the machine learning models that employ mathematical methodologies to enhance prediction accuracy. Logistic regression uses a sigmoid function to model binary outcomes. The random forest aggregates decision trees and employs criteria such as the Gini index and entropy to assess node impurities. Support vector machines (SVM) optimize the margin between classes and use the kernel trick to capture intricate, non-linear patterns. Gradient boosting machines (GBM) systematically improve predictions by minimizing the loss function through iterative learning. Neural networks analyze data using weighted layers and employ activation functions and gradient descent for optimization.

### Estimating AIC/BIC for machine learning models

Because the traditional akaike information criterion (AIC) and bayesian information criterion (BIC) rely on likelihood functions, which most machine learning models lack, we used an approximation based on the model’s loss function. Specifically, we employed log loss (cross-entropy) to estimate the negative log-likelihood for classification-based survival predictions^37^. The log-likelihood is approximated as follows:

$$\:\text{log}\mathcal{L}\approx\:-n\times\:\text{Log-Loss}$$

Where n is the number of observations. Using this, the AIC and BIC were computed as follows:

$$\:\text{AIC}=2k-2\text{log}\mathcal{L},\quad\:\text{BIC}=k\text{log}\left(n\right)-2\text{log}\mathcal{L}$$

Here, k refers to the effective number of the model parameters.

## Experimental results

### Effect of demographic and clinical factors on survival probability

Table 4 presents the coefficients and p-values of various demographic and clinical variables across six parametric survival models: Weibull, Exponential, Gaussian, Logistic, Log-logistic, and Log-Gaussian.

- **Age**: Demonstrated a statistically significant positive correlation with survival in the weibull and log-gaussian models (*p* < 0.05), suggesting that advancing age is associated with a higher probability of survival.
- **Tumor grade**: Exhibited a significant negative correlation with survival in the Weibull model, indicating that elevated tumor grades reduce survival probability.
- **Primary tumor site**: Demonstrated statistical significance exclusively in the weibull model, while other models did not yield conclusive associations, indicating minimal influence on survival estimates.
- **Marital status** exhibited statistical significance in the weibull model, indicating an association between marital status and improved survival outcomes.
- **AJCC stage**: Demonstrated a statistically significant negative correlation with survival in the Weibull model, indicating that patients with advanced-stage cancer have a reduced survival probability. The exponential model was statistically significant (*p* = 0.04).
- **Race**: Significantly associated with survival outcomes in the weibull model but lacking robust statistical evidence in other parametric models.
- **Radiation therapy**: demonstrated statistical significance across multiple survival models, including weibull, gaussian, logistic, log-logistic, and log-gaussian models, underscoring its potential impact on survival outcomes.
- **Chemotherapy**: Attained statistical significance in the weibull model, indicating that its association with survival varied depending on the assumed parametric distribution.

**Table 4:** Regression coefficients and P-values for various parametric survival Models.

Variables	Weibull	Exponential	Gaussian	Logistic	Log-Logistic (e-05)	Log-Gaussian
Age	0.01044 (*p* < 2e-16)	0.0026 (*p* = 0.94)	0.666 (*p* = 0.044)	0.2973 (*p* = 0.17)	4.60e-03 (*p* = 0.27)	0.020128 (*p* = 0.035)
Grade	−0.03753 (*p* < 2e-16)	0.0112 (*p* = 0.63)	−0.1646 (*p* = 0.413)	−0.1180 (*p* = 0.37)	−1.84e-03 (*p* = 0.47)	−0.002423 (*p* = 0.67)
Primary Site	−0.01319 (*p* < 2e-16)	0.0088 (*p* = 0.44)	0.0276 (*p* = 0.78)	−0.0017 (*p* = 0.97)	4.48e-05 (*p* = 0.97)	0.001903 (*p* = 0.50)
Marital Status	−0.04215 (*p* < 2e-16)	0.0437 (*p* = 0.11)	0.2864 (*p* = 0.220)	0.1868 (*p* = 0.22)	3.08e-03 (*p* = 0.29)	0.011280 (*p* = 0.09)
AJCC Stage	−0.17032 (*p* < 2e-16)	0.0918 (*p* = 0.04)	−0.0287 (*p* = 0.94)	−0.1452 (*p* = 0.57)	−1.58e-03 (*p* = 0.75)	0.000678 (*p* = 0.953)
Race	−0.00646 (*p* < 2e-16)	−0.0149 (*p* = 0.71)	−0.6764 (*p* = 0.052)	−0.2703 (*p* = 0.24)	−4.71e-03 (*p* = 0.29)	−0.018576 (*p* = 0.06)
Radiation	−0.03558 (*p* < 2e-16)	0.0032 (*p* = 0.75)	−0.3894 (*p* = 6.3e-06)	−0.1625 (*p* = 0.004)	−2.77e-03 (*p* = 0.01)	−0.010289 (*p* = 3.6e-05)
Chemotherapy	0.02296 (*p* < 2e-16)	−0.0093 (*p* = 0.86)	0.0135 (*p* = 0.977)	0.1238 (*p* = 0.69)	1.70e-03 (*p* = 0.77)	−0.002486 (*p* = 0.856)

**Fig. 2:**
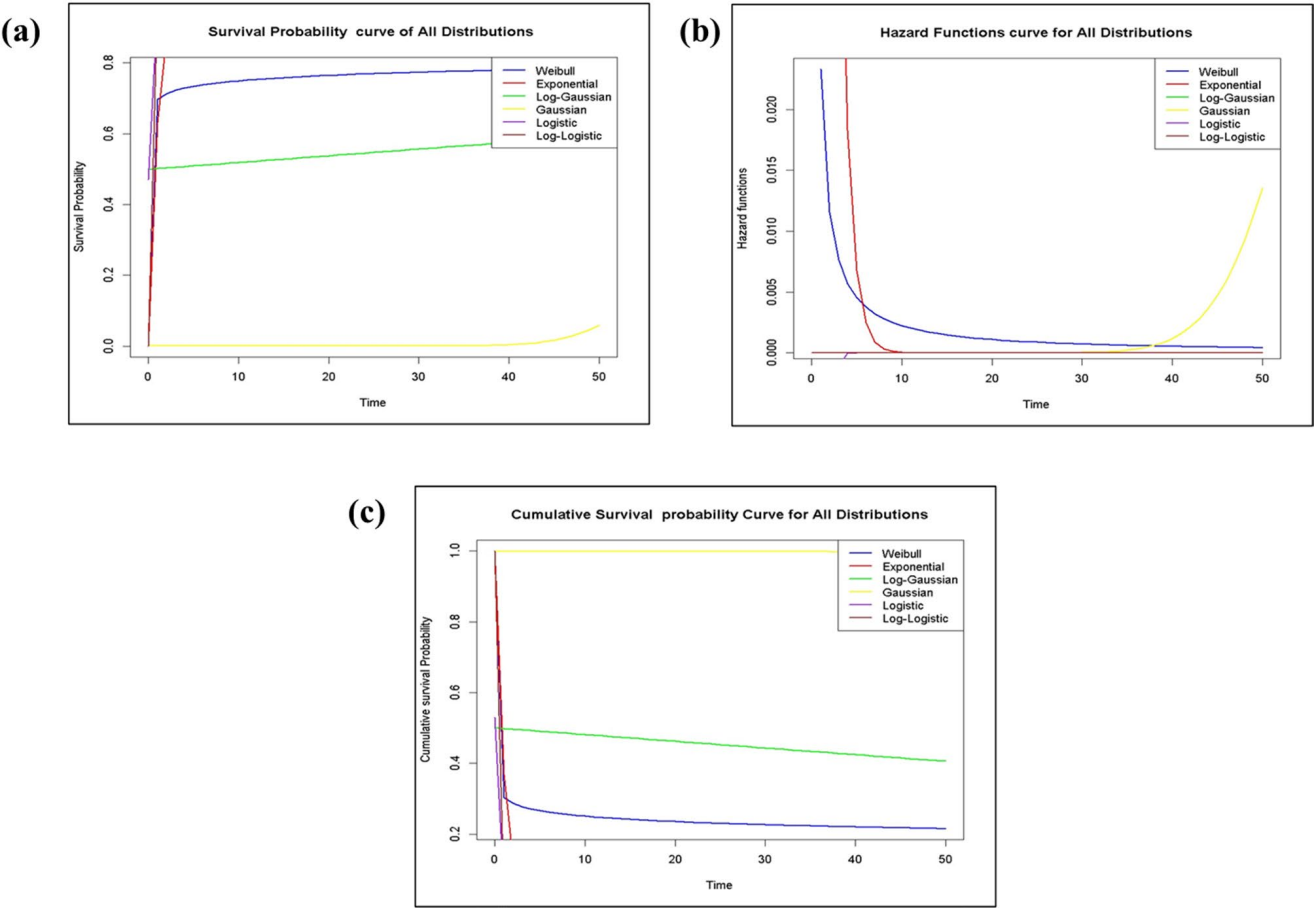
(**a**) Survival Probability, (**b**) Cumulative Survival, and (**c**) Hazard Function Curves for Parametric Survival Distributions.

Figure 2 illustrates the survival probability, hazard function, and cumulative survival probability for the six parametric models: weibull, exponential, gaussian, logistic, log-gaussian, and log-logistic. The survival probability curve depicts the variation in survival likelihood over time across various distributions. The weibull and log-logistic functions exhibited rapid decreases, followed by stabilization, whereas the exponential function remained consistently low. Log-gaussian and gaussian distributions show a more gradual decline, indicating long-term survival patterns. The hazard function curve illustrates that the weibull risk decreases over time, the exponential model maintains a constant failure rate, and the log-gaussian models show an increasing hazard over time. The logistic and log-logistic models demonstrated an initially elevated risk that diminished as time progressed.

The cumulative survival probability curve (representing the cumulative failure probability) shows the escalation of failure risk over time for various distributions. The weibull and log-logistic functions displayed an initially steep increase, whereas the exponential model remained stable. In contrast, the log-gaussian and gaussian models exhibit a progressive increase, signifying prolonged longevity.

### Evaluation of model fit using AIC and BIC

The akaike information criterion (AIC) and bayesian information criterion (BIC) in Table 5 evaluate the model fit by balancing goodness-of-fit with complexity, with lower values signifying better models. This investigation revealed that among all the evaluated models, the random forest approach exhibited the best performance, as evidenced by the lowest AIC (568.70) and BIC (1274.49) values, signifying a significant balance between model fit and complexity. Within the context of standard survival models, the exponential distribution was revealed to be the most effective, obtaining notably lower AIC (17,445.14) and BIC (17,495.84) values than alternative distributions such as weibull, gaussian, and log-logistic. In contrast, the support vector machine (SVM) exhibited exceptionally high AIC and BIC values.

**Table 5:** Comparative evaluation of parametric and machine learning models for survival prediction using AIC and BIC.

Model	Parametric		Machine Learning		
	AIC	BIC	Model	AIC	BIC
Weibull	99012.44	99068.77	Logistic Regression	3445.55	10204.18
Exponential	17445.14	17495.84	SVM	434782.21	1,310,526
Gaussian	11983.49	12039.82	Gradient Boosting	16,318	47272.56
Logistic	11113.49	11169.82	Neural Network	20151.03	60574.65
Log-Logistic	12012.69	12069.03	Random Forest	568.70	1274.49
Log-Gaussian	13974.82	14031.16			

### Performance comparison of machine learning models

Table 6 presents a comparative analysis of the machine learning models, specifically logistic regression, random forest, support vector machine, gradient boosting machine, and neural network, based on the accuracy, precision, recall, F1-score, and AUC. The neural network demonstrated the highest overall performance, with an accuracy, precision of 0.815, recall of 0.984, and an F1-score of 0.809, 0.815, 0.984, and 0.982, respectively, indicating that it was the best predictive model for breast cancer. Logistic regression followed closely, with similar accuracy (0.808), precision (0.815), and recall (0.982). The gradient boosting machine (GBM) recorded the highest AUC (0.656), demonstrating superior class separation, although its accuracy (0.798) and precision (0.809) were slightly lower. The support vector machine (SVM) performed well in terms of recall (0.982) but had the lowest AUC (0.608), suggesting a less predictive model. Random forest underperforms, with the lowest accuracy (0.769) and F1-score (0.865), indicating a weaker trade-off between precision and recall. Although neural networks are the most well-rounded, logistic regression offers simplicity and interpretability, and the GBM excels in classification ranking. The optimal model depends on the application and whether it prioritizes interpretability, sensitivity, or ranking performance.

**Table 6:** Classification performance of predictive models.

Model	Accuracy	Precision	Recall	F1 Score	AUC
Logistic Regression	0.808	0.815	0.982	0.891	0.645
Random Forest	0.769	0.812	0.926	0.865	0.619
SVM	0.795	0.804	0.982	0.884	0.608
GBM	0.798	0.809	0.978	0.885	0.656
Neural Network	0.809	0.815	0.984	0.982	0.645

**Fig. 3:**
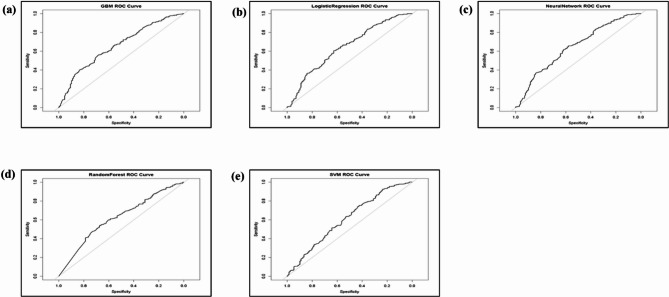
ROC Curve Illustrating Performance of **(a)** GBM, **(b)** LR, **(c)** Neural Network, **(d)** RF, and **(e)** SVM.

The ROC curve, which represents the logistic model, is shown in Fig. 3. The neural network is the best predictive model. The ROC curves illustrate the balance between sensitivity (true positive rate) and specificity (1–false positive rate) for each model. The closeness of the curves to the diagonal indicates that all models possessed limited discriminatory power, possibly with AUC values ranging from 0.6 to 0.7. AUC values around 0.5 signify random performance, and values near 1 imply robust classification capability. The models demonstrated no substantial superiority, indicating similar prediction efficacy.

### Performance comparison of machine learning models

Our models exhibit competitive and balanced performance in comparison to previous studies. Neural network and logistic regression attained accuracies of 80.9% and 80.8%, respectively, with elevated recall values (up to 0.984) and F1-scores (up to 0.982), signifying robust predictive performance. Although prior studies, including Ahmed et al. (2025) and Nurul Amirah Mashudi et al. (2020), documented better accuracies of 98.57% and 98.60% utilizing random forest and SVM, respectively, our models demonstrate enhanced performance compared to Reza Rabiei et al. (2022), whose random forest model attained an accuracy of 80%. Our findings underscore consistent performance across many evaluation metrics, illustrating the efficacy of the employed models in breast cancer prediction. Most prior research on breast cancer prediction works on different datasets and does not explicitly address invasive lobular carcinoma (ILC). As indicated in Table 7, models were utilized widely without identifying ILC as a separate subtype. This work bridges the gap by concentrating solely on ILC, offering more precise insights and prediction outcomes relevant to this underexplored and clinically significant breast cancer subtype.

**Table 7:** Comparison of breast cancer prediction accuracy across previous Studies.

*R*. No	Author	Year	Data Set	Methods	Results (Accuracy)
^37^	Ahmed et al.	2025	SEER database	RF	98.57
^38^	Islam T et al.	2024	SEER breast cancer database	DT	91%
^39^	Taminul Islam et al.	2024	Breast Cancer Primary Dataset	XGBoost	97%
^40^	Varsha Nemade et al.	2023	Wisconsin Diagnostic Breast Cancer (WDBC) Dataset	XGBoost	97%
^38^	P. Manikandan et al.	2023	SEER breast cancer dataset	DT	98%
^41^	Reza Rabiei et al.	2022	Motamed Cancer Institute (ACECR), Tehran, Iran	RF	80%
^42^	Nurul Amirah Mashudi et al.	2020	WDBC Dataset	SVM	98.60%

**Strength:**

1. Focus on invasive lobular carcinoma (ILC): Addresses a significant gap by targeting a less-studied breast cancer subtype, enhancing clinical relevance.
2. Extensive parametric model comparison: Applied a broader set of parametric models than typically used, going beyond cox and standard forms to include multiple distributions for a thorough survival analysis.
3. AIC/BIC applied to ML models using log-loss: Innovatively applied AIC and BIC to machine learning models by approximating likelihood through log-loss and pseudo-likelihood methods, enabling a unified model evaluation framework.

## Conclusion

This study evaluated the predictive performance of both parametric and machine learning models in estimating survival outcomes among patients with invasive lobular carcinoma. Several key prognostic factors, including age, tumor grade, ajcc stage, marital status, and radiation therapy, were found to significantly influence survival. The performance of the models varied depending on the evaluation criteria used. Neural networks showed relatively higher predictive accuracy when assessed using classification metrics such as the area under the receiver operating characteristic curve (AUC) and precision. In contrast, when evaluated using information-based criteria that focus on model fit while penalizing complexity, the random forest model performed best, as indicated by the lowest values for the akaike information criterion (AIC) and the bayesian information criterion (BIC). These results highlight the tradeoffs between accuracy-driven and complexity-aware evaluation methods, emphasizing the importance of using multiple metrics to assess survival models effectively.

Despite these findings, several limitations impact the generalizability and practical use of the study. The SEER database lacks several detailed clinical variables, such as recurrence status, surgical margin information, and data on postoperative complications, all of which could affect survival predictions. Moreover, although some machine learning models outperformed others, their AUC values remained moderate, ranging from 0.60 to 0.66, indicating limited ability to distinguish between outcomes. Parametric models are limited by strict assumptions regarding the underlying data distribution, whereas machine learning models may encounter challenges such as overfitting, limited interpretability, and substantial computational requirements. Moreover, exclusive reliance on selection criteria such as the akaike information criterion (AIC) and the bayesian information criterion (BIC) may bias model selection toward simpler structures, potentially compromising predictive performance.

In summary, this research shows the value of combining statistical and machine learning approaches in cancer survival prediction. These methods offer complementary strengths interpretability from parametric models and flexibility from machine learning techniques. However, developing clinically useful models will require access to more detailed, diverse datasets and continued methodological improvements. Future research should focus on hybrid modeling techniques that bring together the strengths of both approaches to better capture complex survival patterns. Enhancing methods for handling censored data, which is common in survival studies, will improve the accuracy and reliability of predictions. Including time-varying variables could also provide a more accurate picture of changes in a patient’s condition or treatment over time, leading to more relevant and dynamic models. Lastly, expanding evaluation metrics beyond AIC and BIC would allow for a more balanced and comprehensive assessment of model performance.

## Data Availability

The analysis was based on publicly accessible secondary data from the https://seer.cancer.govdatabase.
